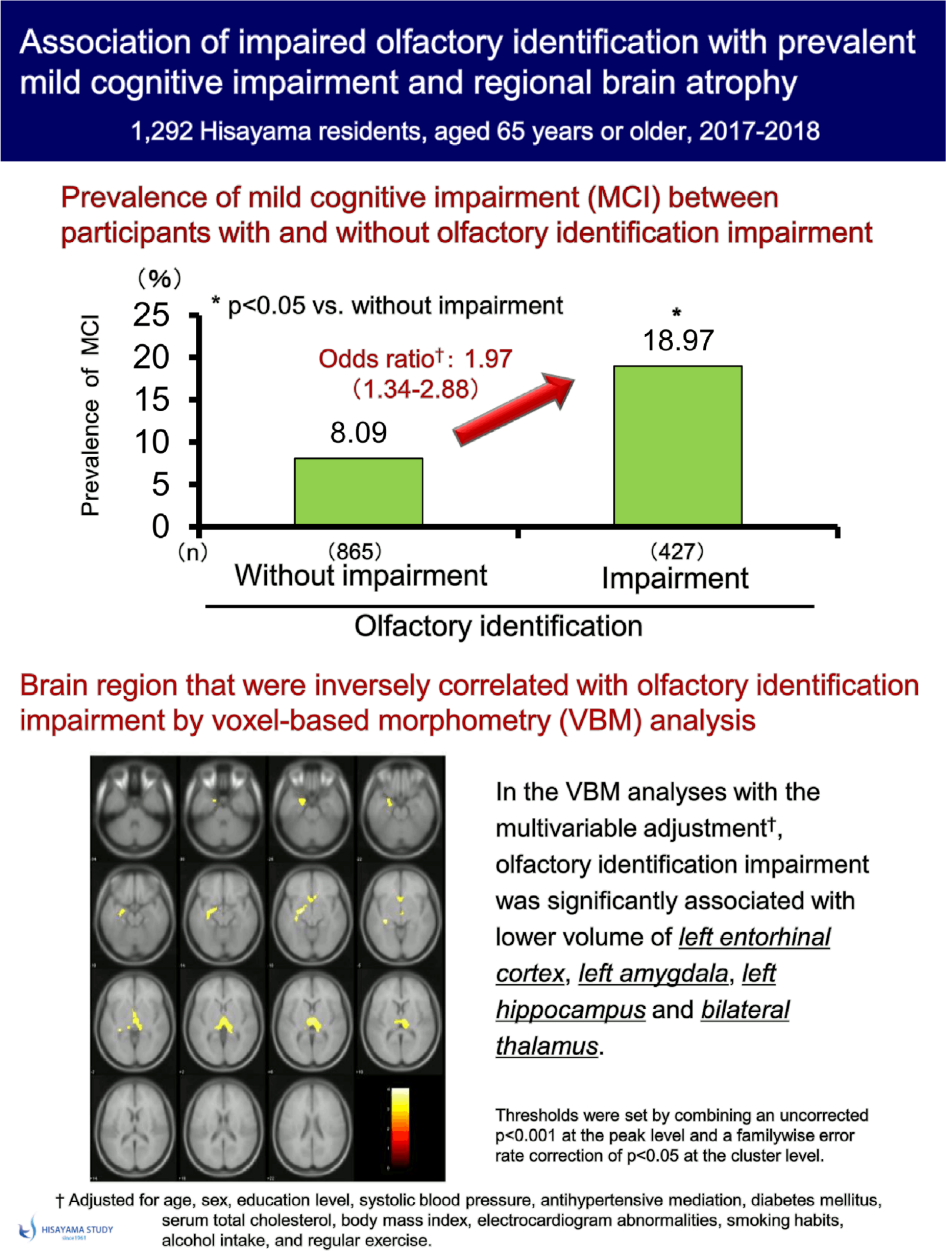# Association of impaired olfactory identification with prevalent mild cognitive impairment and regional brain atrophy: the Hisayama Study

**DOI:** 10.1002/alz.089757

**Published:** 2025-01-09

**Authors:** Taro Nakazawa, Tomoyuki Ohara, Toshifumi Minohara, Jun Hata, Mao Shibata, Toshiharu Ninomiya

**Affiliations:** ^1^ Graduate School of Medical Sciences, Kyushu University, Fukuoka Japan

## Abstract

**Background:**

Few population‐based studies have comprehensively examined the association between impaired olfactory identification and the prevalence of mild cognitive impairment (MCI) and regional brain volumes in a general older population without dementia.

**Method:**

A total of 1,292 participants without dementia aged 65 years or older underwent a Japanese Pocket Smell Test, an assessment of cognitive function, and a brain magnetic resonance imaging scanning in 2017‐2018. Impaired olfactory identification was defined as incorrect identification of three or more odors in the Japanese Pocket Smell Test, where participants sniffed eight microencapsulated odors (strawberries, chocolate, mint, smoke, soap, grapes, onions, and roses) and selected the corresponding odor from four choices for each odor. The association of impaired olfactory identification with prevalent MCI was estimated by a logistic regression analysis. Regional brain volumes were estimated separately by applying two different Methods: voxel‐based morphometry (VBM) and analysis by FreeSurfer software. The association of impaired olfactory identification with regional gray matter volumes was analyzed using multiple regression analysis.

**Result:**

Out of 1,292 participants, 427 (33.05%) had an impaired olfactory identification. The crude prevalence of MCI in participants with and without impaired olfactory identification was 18.97% and 8.09%, respectively. Participants with impaired olfactory identification had a significantly higher likelihood of MCI than those without after adjusting for confounders (odds ratio 1.97, 95% confidence intervals 1.34–2.88). In the FreeSurfer analysis, impaired olfactory identification was significantly associated with lower gray matter volume of entorhinal cortex, inferior temporal gyrus, amygdala, thalamus, cingulate, parahippocampal gyrus, and hippocampus. The sensitivity analysis using multivariable‐adjusted VBM analysis without regions of interest also showed that impaired olfactory identification was significantly associated with lower volume of left entorhinal cortex, left amygdala, left hippocampus, and bilateral thalamus.

**Conclusion:**

Our findings suggest that the assessment of olfactory identification may be a noninvasive and convenient method for identifying high‐risk individuals with cognitive impairment, including neurodegeneration.